# Current Knowledge of Hypertrophic Cardiomyopathy Among Health Care Providers in Sweden

**DOI:** 10.7759/cureus.12220

**Published:** 2020-12-22

**Authors:** Peter Magnusson, Stellan Mörner

**Affiliations:** 1 Center of Research and Development, Uppsala University/ Region Gävleborg, Gävle, SWE; 2 Medicine, Cardiology Research Unit, Karolinska Institutet, Stockholm, SWE; 3 Department of Public Health and Clinical Medicine, Umeå University, Umeå, SWE

**Keywords:** arrhythmia, hypertrophic cardiomyopathy, sudden cardiac death

## Abstract

Introduction

Hypertrophic cardiomyopathy (HCM) is a common disorder with various manifestations, including sudden cardiac death. Patients with suspected or confirmed HCM may be encountered throughout the healthcare system, especially in internal medicine and cardiology. Thus, thorough knowledge of HCM is essential among healthcare providers.

Methods

A web-based questionnaire was developed to assess the cross-sectional evaluation of HCM knowledge. It covered aspects such as epidemiology and diagnosis, treatment, lifestyle, risk stratification of sudden cardiac death, and implantable cardioverter-defibrillator knowledge.

Results

In total, 123 subjects completed the survey. The mean age was 38.5 ±10.7 years and two-thirds (n=82) were females; 43.1% were physicians (non-specialist 24.4%, cardiologists 8.9%, specialist, other than cardiology 9.8%); and the remaining were nurses (nurses within cardiology 37.4%, nurses outside cardiology 19.5%). Almost all subjects had heard about the disease (95.9%) and the vast majority (77.2%) had taken part in the management of a patient with HCM. The total mean score was 15.9 ±3.9 credits and the 25th, 50th, and 75th percentiles were 14, 15, and 18 credits, respectively. The predefined arbitrary pass score of ≥60% was reached by 61.8%, and 20.3% were considered to pass with distinction. Physicians scored higher than nurses (70.7 ±17.0% vs 58.1 ±11.8; p<0.001). Within each professional category, there was a similar score with regard to gender.

Conclusions

There is a considerable lack of knowledge of HCM among healthcare professionals working within the field of internal medicine/cardiology. This insufficient knowledge may contribute to less implementation of evidence-based medicine and current guidelines, although further studies are needed to confirm this.

## Introduction

The hypertrophic cardiomyopathy (HCM) phenotype in adults is characterized by at least 15 mm wall thickness of any myocardial segment, which is not explained by other myocardial diseases or abnormal loading conditions such as hypertension or aortic valve stenosis [1]. About half of HCM patients have a sarcomere gene mutation that explains the disease, which can be useful in cascade screening of family members [2-3]. In the general population, approximately 1:500 individuals fulfill the criteria for HCM [4-6]. Most symptomatic patients suffer from exercise intolerance with dyspnea, chest discomfort, palpitations, and dizziness. The most prominent segment of hypertrophy is typically localized in the septum and may affect the left ventricular outflow tract (LVOT), causing obstruction of the blood flow that can be quantified as a gradient [7]. Sometimes, systolic anterior motion (SAM) of the mitral valve is encountered. Echocardiography is a cornerstone in the diagnostic workup but cardiac magnetic resonance (CMR) should be performed and may provide a more accurate assessment, be useful for differential diagnosis, and potentially provide information for risk stratification [8].

Sudden cardiac death (SCD) risk stratification and recommendations of an implantable cardioverter-defibrillator (ICD) is based on current guidelines. In the 2011 American ACCF/AHA guidelines, the presence of non-sustained ventricular tachycardia, maximum wall thickness ≥30 mm, unexplained syncope, a history of SCD among first-degree relatives, and blood pressure response during exercise constitute risk factors [9]. In the European guidelines from 2014, an algorithm has been endorsed that takes several factors into account: non-sustained ventricular tachycardia (NSVT), left atrial diameter, unexplained syncope, maximum wall thickness, LVOT gradient, family history of SCD but also age [10-12]. Notably, according to this algorithm, the risk of SCD increases with lower age. A five-year SCD risk of ≥6% implies the recommendation of an ICD (class IIa indication), ≥4% is considered a less strong indication (class IIb) [12].

In HCM, atrial fibrillation (AF) is known to worsen symptoms due to particular vulnerability to increased heart rate and lack of atrial filling. AF is common in HCM and because of the high risk of embolization, anticoagulation is recommended regardless of the CHA2DS2-VASc score [12-13].

There is no proven pharmaceutical agent to reduce hypertrophy but beta-blockers are generally prescribed in symptomatic patients [14]. Angiotensin-converting enzyme/angiotensin receptor blocker (ACE/ARB) can be useful in patients with concomitant hypertension but its afterload reducing properties may be a particular concern in those with a significant LVOT gradient [12]. Instead, in highly symptomatic patients with septal hypertrophy, myectomy or alcohol septal ablation effectively alleviates symptoms due to obstruction [15]. In patients who already have a pacemaker or ICD, apical right ventricular pacing can cause dyssynchrony, which may reduce obstruction but is nowadays not part of routine care [12].

Living with HCM often requires some lifestyle modifications [12,16]. Intense athletic sports activity should be avoided [17]. Pregnancy is generally not contraindicated but counseling in individual cases is advocated [18]. Depending on the severity of the disease, anesthetic precautions should be considered. In general, endocarditis prophylaxis is not indicated [19].

Because HCM is a common disease, knowledge among healthcare providers is warranted. Among healthcare providers, who are active as clinicians, in the internal medicine/cardiology units, a thorough understanding of disease management could be expected. However, the level of knowledge of HCM among physicians and nurses serving in clinical internal medicine/cardiology clinics is unknown.

## Materials and methods

The questionnaire

The questionnaire was developed by the authors with the intention to assess the cross-sectional evaluation of HCM knowledge. The questionnaire was divided into two parts and each question (Q) had one correct answer; part A was multiple-choice (five alternatives) and part B contained statements that were either true or false. The predefined pass score was ≥60% and 80% was considered as pass with distinction. The overall principle was to ascertain knowledge with clinical relevance based on guidelines on HCM and with a focus on risk stratification with regard to SCD. We created 25 questions within the following domains: Epidemiology and diagnosis (Q 1, 2, 3, 4, 5, 9, 10, 13, 14), Treatment (Q 6, 11, 12, 15, 16), Life-style (Q 17, 18, 19), Risk stratification of SCD (Q 7, 8, 20, 21, 22), and ICD/pacemaker technology knowledge (Q 23, 24, 25).

The pilot test comprised 12 non-specialist physicians after which minor modifications were done for clarity. Next, the questionnaire was distributed by e-mail (including two reminders) with a link to a web page to ensure the completeness of the answers. In total, approximately 420 professionals received the e-mail.

Setting

These professionals were physicians and nurses currently working within the field of cardiology or internal medicine in Region Gävleborg in Sweden (the three hospitals Bollnäs, Gävle, and Hudiksvall). The physicians were divided into three categories: Cardiologist; Physician, specialist; and Physician, not specialist while the nurses were divided into two categories: Nurse, cardiology; Nurse, non-cardiology.

Statistical analyses

Data were described as numbers (n), percentages, ranges, percentiles, means, and standard deviations. To analyze the association between variables, the chi-squared test, and the t-test as appropriate. A two-sided p-value <0.05 was considered significant. The Statistical Package for the Social Sciences (SPSS) version 25 (IBM Corp., Armonk, NY) was used for statistical analyses.

Ethics

To ensure confidentiality, the name of the participants were replaced by a code blinded for the investigators. Each participant was informed that the survey was voluntary and approved it in written form. Ethics approval was deemed unnecessary according to national regulations.

## Results

In total, 123 subjects completed the survey. The mean age was 38.5 ±10.7 years; the 25th, 50th, and 75th percentiles were 23, 30, and 47 years, respectively. Two-thirds (n=82) were females. In the sample, 43.1% were physicians (non-specialist 24.4%, cardiologist 8.9%, specialist, other than cardiology 9.8%) and the remaining were nurses (nurses within cardiology 37.4%, nurses outside cardiology 19.5%). Among physicians, 73.2% (n=30) were males and among nurses, a majority were females (84.3 %, n=59). The professional experience, expressed as time since exam year showed a skewed distribution: the 25th, 50th, and 75th percentiles were three, nine, and 19 years, respectively.

Almost all subjects had heard about the disease (95.9%), and the vast majority (77.2%) had taken part in the management of a patient with HCM.

**Figure 1:**
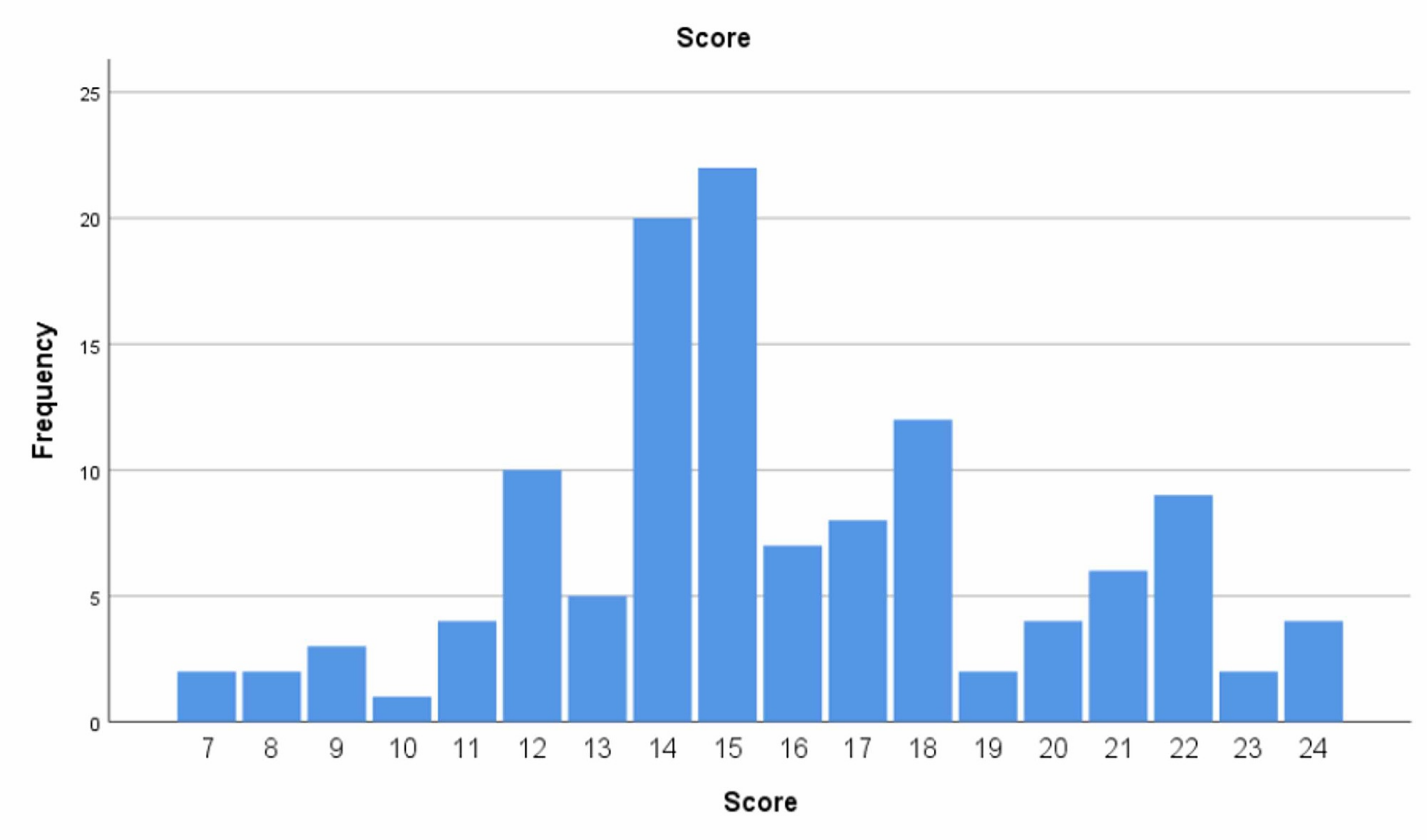
The survey “Knowledge about hypertrophic cardiomyopathy,” distribution of total score

Each question and the answers, including the distribution, are summarized in Table 1, Table 2, and Figure 1. Based on the number of alternatives, a pure guess would yield a score of 10.1 out of 25.

**Table 1:** The survey “Knowledge about hypertrophic cardiomyopathy,” Part 1 Each question/statement is followed by five answers of which one is correct. The distribution between the answers among the subjects is presented as percentages. Physicians percentage of correct answers within parenthesis.

Nr	Question	A	B	C	D	E
1	What is the prevalence of HCM in the general population?	1:5	1:50	1:500	1:5000	1:50000
	Correct: C	1.6%	5.7%	43.9% (54.7%)	32.5%	16.3%
2	Which is the wall thickness required for the diagnosis of HCM in adults?	At least 9 mm	At least 12 mm	At least 15 mm	At least 17 mm	At least 20 mm
	Correct: C	1.6%	5.7%	67.5% (71.7%)	18.7%	6.5%
3	Myocardial hypertrophy may be caused by all EXCEPT…	Aortic stenosis	Aortic insufficiency	Hypertension	Amyloidosis	Anderson-Fabry
	Correct: B	2.4%	46.3% (66.0%)	7.3%	17.1%	26.8%
4	Typical echocardiographical findings in HCM may include the following EXCEPT…	Septal hypertrophy	Increased left ventricular outflow tract gradient	SAM (systolic anterior motion) of the mitral valvue	Apical hypertrophy	Pulmonary stenosis
	Correct: E	4.1%	12.2%	20.3%	4.9%	58.5% (69.8%)
5	One of the following exams is NOT routinely considered in HCM…	24-48 hour ECG (Holter)	Echocardiography	Cardiac-MR	Genetic test	Myocardial biopsy
	Correct: E	19.5%	0%	9.8%	12.2%	58.5% (75.5%)
6	Which pharmacological agent is first-line in symptomatic HCM?	Metoprolol	Verapamil	Disopyramide	Amiodarone	Dronedarone
	Correct: A	72.4% (83.0%)	17.9%	3.3%	5.7%	0.8%
7	In the American guidelines (2011) of HCM the following risk factors for sudden cardiac death are included EXCEPT…	Non-sustained ventricular tachycardia	Maximum myocardial hypertrophy ≥ 30 mm	Unexplained syncope	ECG-criteria	Family history of sudden cardiac death (child, parent, sibling)
	Correct: D	26.8%	5.7%	17.1%	43.9% (64.2%)	6.5%
8	In the European guidelines (2014) of HCM the following variables are included in the risk calculator EXCEPT…	Age	Unexplained syncope	Maximum left myocardial wall thickness	Abnormal blood pressure response	Left ventricular outflow gradient
	Correct: D	35.8%	8.9%	7.3%	34.1% (30.2%)	13.8%

**Table 2:** The survey “Knowledge about hypertrophic cardiomyopathy,” Part 2 Each statement is either true or false. The correct answer highlighted. The distribution between the answers among the subjects is presented as percentages. Physicians percentage of correct answers within parenthesis.

Nr	Question	True	False	Physicians
9	The diagnosis of HCM requires a mutation that explains the disease.	10.6%	89.4%	(94.3%)
10	In HCM is the increased thickness most often localized to the septum even though other localizations do occur, i.e. apical.	80.5%	19.5%	(75.5%)
11	ACE/ARB-drugs are always indicated in HCM.	48.8%	51.2%	(71.7%)
12	Anticoagulation is indicated in HCM patients with atrial fibrillation regardless of CHADSVASC-score.	72.4%	27.6%	(56.6%)
13	In index patients with HCM, but without mutation, first degree family members do NOT need evaluation with echocardiography.	43.9%	56.1%	(66.0%)
14	Cascade screening is indicated in relatives of a patient with genetically verified HCM.	91.1%	8.9%	(94.3%)
15	Septal reduction intervention can be performed by surgical myectomy or alcohol ablation.	73.2%	26.8%	(84.9%)
16	Pacemaker is routinely indicated to treat symptomatic obstruction (of the left ventricular outflow tract).	28.5%	71.5%	(79.2%)
17	HCM patients should abstain from competitive soccer.	86.2%	13.8%	(90.6%)
18	Endocarditis prophylaxis is always indicated in HCM.	14.6%	85.4%	(92.5%)
19	Pregnancy is generally contraindicated in HCM.	43.1%	56.9%	(73.6%)
20	In European guidelines from 2014, increased age and larger left atrial diameter imply a higher 5-year risk of sudden cardiac death.	76.4%	23.6%	(32.1%)
21	In European guidelines from 2014, in HCM patients with a 5-year risk of ≥6% an ICD is indicated (Class IIb).	86.2%	13.8%	(94.3%)
22	In European guidelines from 2014, in HCM patients with a 5-year risk ≥4% and <6% an ICD may be considered (Class IIb).	69.9%	30.1%	(34.0%)
23	When a magnet is placed on an ICD, treatment of ventricular tachycardia is inactivated (shock and antitachycardia pacing).	85.4%	14.6%	(83.0%)
24	When a magnet is placed on an ICD, the pacing function is inactivated.	35.0%	65.0%	(67.9%)
25	When a magnet is placed on a pacemaker, the pacing function is inactivated.	39.8%	60.2%	(62.3%)

The total score was mean 15.9±3.9 and the 25th, 50th, and 75th percentiles were 14, 15, and 18 credits, respectively. Physicians scored higher than nurses (70.7 ±17.0% vs 58.1 ±11.8; p<0.001). Among physicians, females and males had similar scores (71.5 ±17.0% vs 69.8 ±16.0%; p=0.714). Similar scores were also seen among female and male nurses, respectively (55.9 ±11.5% vs 59.5 ±10.7%; p=0.319). The mean scores with regard to different professions are depicted in Table 3.

**Table 3:** The survey “Knowledge about hypertrophic cardiomyopathy,” total score with regard to professional categories

Category	n	Total score	60% of Total score (pass) n=76 (61.8%)	80% of Total score (pass w distinction) n=25 (20.3%)
Cardiologist	11	84.4 ±18.2%	10	10
Physician, specialist	12	72.0 ±13.8%	10	4
Physician, not specialist	30	65.3 ±15.4%	23	7
Nurse, cardiology	46	60.6 ±13.8%	25	4
Nurse, non-cardiology	24	53.3 ±8.9%	8	0

## Discussion

Implementation of scientific guidelines is of utmost importance to provide current evidence-based medicine to patients. Nevertheless, knowledge among clinically active professionals is seldom measured. HCM may be encountered in various settings within clinical medicine but professionals within internal medicine/cardiology need thorough knowledge within the field.

As expected cardiologists scored higher than the other professional categories. Moreover, physicians scored higher than nurses but the difference between physicians without specialization and nurses working in a cardiology unit was small. The score was similar between genders in each category. The predefined arbitrary pass score of ≥60% was reached by 61.8% and 20.3% was considered as pass with distinction.

Epidemiology and diagnosis

The basic epidemiology, i.e. prevalence of HCM in the general population, which is often cited in the literature, was only known by approximately half the participants. From an individual management perspective, the 15 mm cut-off for diagnosis is elementary knowledge for healthcare professionals who interpret the imaging evaluations of potential HCM patients. This gap of knowledge is repeated in Q3 and Q4 and stresses the importance that both referrals and examiners of echocardiography are aware of elaborative evaluations that guide clinicians to manage the patients with referrals to HCM-specialists when appropriate. The same holds true as a fifth of the participants did not recognize the routine use of ambulatory Holter monitoring in HCM patients. Luckily, the vast majority correctly answered the question about cascade screening of relatives when an index patient is genopositive. However, a lack of basic understanding of the inheritance pattern or the fact that genopositivity is not a prerequisite for diagnosis is revealed by the answers to Q13 and 14. Indeed, a third of physicians were not aware that genopositivity is not compulsory for diagnosis, and this may lead to neglect or improper management of families with continued follow-up with echocardiography when there is no disease explaining mutation.

Treatment

The vast majority correctly answered metoprolol as the first-line pharmacological therapy and most other participants answered verapamil, which is a second-line choice. ACE/ARB may be indicated in concomitant hypertension or systolic heart failure but should be avoided in patients with significant left ventricular outflow gradients due to its afterloading properties. Even though physicians were more knowledgeable about this, there was a knowledge gap. The reverse, i.e. physicians score worse on the question about anticoagulation in HCM patients with a history of AF; they were not aware of the exception to the general rule that at least one CHADSVASC risk factor, is required. The majority seem to be aware of the surgical myectomy and alcohol septal ablation in order to reduce outflow tract obstruction; although a considerable knowledge gap concerning the management of HCM was assessed.

Lifestyle

The association of SCD and HCM in athletes is occasionally a topic of interest by mainstream media. This may explain the high awareness about recommendations to abstain from competitive sports. Similarly, the participants seem to know that endocarditis prophylaxis is not advised in HCM in general. On the contrary, healthcare professionals may have a view that HCM is generally a disease with constraints, as many thought pregnancy to be generally contraindicated.

Risk stratification

Risk stratification with regard to SCD is the main task for the routine follow-up of HCM patients. Less than half of the sample, and about two-thirds of physicians, had knowledge of the classic risk factors for SCD. Unfortunately, the majority were not updated about the current algorithm for risk stratification that has been endorsed by the European Society of Cardiology (ESC). Moreover, the interpretation of the ESC risk calculator and cut-offs for ICD recommendation showed considerably low levels of knowledge, even among physicians.

ICD knowledge

The vast majority, and with similar results between nurses and physicians, knew that magnet application temporarily disables discharge from the ICD. In another Swedish study of physicians, 87% of cardiologists knew this [20]. Interestingly, 35% of our participants falsely believed that the pacing function is also deactivated by magnet application of the ICD. Nevertheless, this was better than the sample of physicians in another study [20].

Clinical perspectives

HCM is a heterogeneous and complex disease entity that requires an individual approach grounded in evidence-based medicine. Guidelines provide the basis for the implementation of accurate and reasonably updated management. The disease may be encountered in different settings and specialties, which is why knowledge and alertness are warranted for the broad community of health care professionals. This study shows a wide gap of knowledge in different aspects of HCM management among health care providers in internal medicine and cardiology. It underlines the importance of improving knowledge, possibly by education, diagnostic supporting systems, and awareness. An active role of patient organizations, preferably with collaborations of dedicated HCM-centers may be encouraged. Specialist services should be concentrated in central facilities while less specialist parts of care can be provided by the district cardiology service [21-22]. Interaction between different parts of healthcare and educational systems throughout a professional career is crucial. Further studies on the implementation of guidelines within the field of HCM is encouraged.

Limitations

This study reflects knowledge of HCM in healthcare providers in a context, i.e. the Swedish health care system. It is unknown if those who responded to the survey are representative of those who did not. The strength of the conclusion, and thus the generalizability of findings, are weakened due to the limited response rate. The relevance of each question for each professional category can be criticized; however, the overall finding that there is a considerable gap in knowledge about HCM remains valid.

## Conclusions

There is a considerable lack of knowledge about HCM among healthcare professionals working within the field of internal medicine/cardiology. This insufficient knowledge may contribute to the less implementation of evidence-based medicine and current guidelines. Further studies with higher response rates and across different settings are needed to confirm these findings.
